# Documented Borderline Personality Disorder and EMR Indicators of Potential Under-Recognition in Depressive Outpatients

**DOI:** 10.3390/medicina62061120

**Published:** 2026-06-09

**Authors:** Lifang Dang, Shun Lei Oo, Nahathai Wongpakaran, Awirut Oon-arom, Rewadee Jenraumjit, Justin DeMaranville, Tinakon Wongpakaran

**Affiliations:** 1 Mental Health Program, Multidisciplinary and Interdisciplinary School (MIdS), Chiang Mai University, Chiang Mai 50200, Thailand; lifang_dang@cmu.ac.th (L.D.); shunlei_oo@cmu.ac.th (S.L.O.); nahathai.wongpakaran@cmu.ac.th (N.W.); awirut.oonarom@cmu.ac.th (A.O.-a.); rewadee.w@cmu.ac.th (R.J.); justinross.dem@cmu.ac.th (J.D.); 2 Department of Psychiatry, Faculty of Medicine, Chiang Mai University, Chiang Mai 50200, Thailand; 3 Department of Pharmaceutical Care, Faculty of Pharmacy, Chiang Mai University, Chiang Mai 50200, Thailand

**Keywords:** borderline personality disorder, depressive disorders, under-recognition, electronic medical records, outpatient psychiatry, symptom-based indicator, prescribing-based indicator

## Abstract

*Background and Objectives*: This study aimed to estimate the prevalence of documented borderline personality disorder (BPD) diagnosis and study-defined BPD indicators among adults with depressive disorders in outpatient psychiatric care, and to quantify a subgroup who were indicator-positive but had no documented diagnosis (potential under-recognition). *Materials and Methods*: This retrospective, cross-sectional study included adult outpatients with depressive disorders receiving pharmacological treatment at Maharaj Nakorn Chiang Mai Hospital. BPD status was classified using (1) a documented BPD diagnosis in the electronic medical record (EMR) and (2) study-defined indicators, comprising a symptom-based indicator (documentation of ≥5 DSM-5 BPD criteria) and a prescribing-based indicator (antidepressant treatment with concurrent use of antipsychotics and/or mood stabilizers). Prevalence and overlap across diagnoses and indicators were summarized using a Venn distribution. *Results*: Among 1175 patients, 63 (5.4%) had a documented BPD diagnosis. Using EMR indicators, 84 (7.1%) met the symptom-based indicator and 325 (27.7%) met the prescribing-based indicator. In total, 374 (31.8%) had either a documented BPD diagnosis or at least one indicator, while 801 (68.2%) had neither. Overall, 370 (31.5%) met at least one indicator (symptom-based and/or prescribing-based). Among indicator-positive patients, 311 (84.1%) had no documented BPD diagnosis, representing 26.5% (311/1175) of the total cohort. *Conclusions*: Study-defined EMR indicators flagged a substantial subgroup with potential under-recognition of BPD features in depressive-disorder clinics. These indicators may help prioritize targeted assessment and structured diagnostic evaluation to support access to BPD-informed care and support referral to BPD-informed psychotherapy.

## 1. Introduction

Depressive disorders are common mental health conditions characterized by persistent low mood, reduced energy, and diminished interest or pleasure in daily activities [1]. They can substantially impair emotional, social, and biological functioning and are associated with increased morbidity and mortality [2]. Major depressive disorder (MDD) and persistent depressive disorder (PDD) are common subtypes and contribute substantially to disability worldwide [2,3].

Depressive disorders frequently co-occur with other psychiatric conditions, complicating diagnosis and management. Personality disorders (PDs) are particularly relevant because they shape long-term emotion regulation, impulse control, and interpersonal functioning, influencing the presentation and course of depressive illness. Among PDs, borderline personality disorder (BPD) is of particular clinical importance because it is characterized by affective instability, impulsivity, and interpersonal dysfunction, and is often linked to self-harm and suicidal behaviors [4,5,6]. When depressive disorders and BPD co-occur, clinical presentations are commonly more severe and complex and are associated with poorer outcomes than depression alone [2,7,8,9]. Patients with depression and BPD end up being prescribed several drugs (often three or more), even though evidence suggests that psychotherapy tailored to BPD is more effective [8].

Despite this clinical importance, PDs, and BPD in particular, may be under-recognized or under-documented in routine practice. National service data in Thailand suggest a marked gap between routine diagnostic recording and structured assessment. The 2025 Department of Mental Health report recorded 204,662 cases of depression and 12,428 cases of personality disorder nationally, indicating that PD diagnoses comprised only 6.07% of recorded cases among those with depression [10]. In contrast, a study of outpatients with major depressive disorder using structured clinical interviews reported that 77% of depressed outpatients had at least one comorbid PD, with 60% meeting criteria for two or more PDs (often mixed cluster), and BPD being the most common (20%) [11]. Although these estimates are not directly comparable due to differences in setting and measurement, the discrepancy suggests that comorbid PD features may be frequently missed or not recorded in routine Thai clinical practice.

Possible contributors to under-recognition include symptom overlap between depressive disorders and BPD [12], limited use of structured personality assessment in routine practice, and clinician hesitancy to document a BPD diagnosis because of perceived stigma [13]. In busy outpatient settings, assessments often prioritize acute mood symptoms and risk management, leaving less opportunity to evaluate enduring personality features. Under-recognition may contribute to symptom persistence, elevated suicide risk, greater service utilization, and the burden of ‘difficult-to-treat’ depression [14].

In outpatient care, BPD may be under-recognized because structured personality assessment is not routinely implemented and clinical encounters often prioritize acute symptom relief and risk management [15]. Under-recognition may be especially likely when BPD is primarily associated with crisis presentations such as recurrent suicidal attempts or nonsuicidal self-injury (NSSI), and systematic assessment is not pursued when these features are absent [15]. However, clinically significant BPD can present without recurrent self-harm, and functional impairment may be comparable regardless of self-injury status [15]. Consequently, some patients presenting with depression, anxiety, sleep problems, irritability, or somatic complaints may have substantial BPD features that remain insufficiently recognized and addressed.

Symptom overlaps between depressive disorders and BPD further complicate recognition. Dysphoria, guilt, sleep disturbance, suicidality, irritability, and concentration difficulties can occur in both conditions [6,7]. Mood reactivity and rapid affective shifts may be interpreted as depressive severity, anxiety, or situational stress, rather than prompting an assessment for BPD. As a result, patients with prominent BPD features may be diagnosed and treated primarily as having depressive disorders, contributing to diagnostic overshadowing and incomplete formulation.

The consequences of incomplete recognition are clinically meaningful. Treatment for depressive disorders commonly begins with antidepressant pharmacotherapy such as selective serotonin reuptake inhibitors (SSRIs) and serotonin–norepinephrine reuptake inhibitors (SNRIs). However, antidepressant strategies may be insufficient for core BPD domains such as affective instability, interpersonal dysfunction, and impulsivity [16]. Evidence-based psychotherapies—including dialectical behavior therapy (DBT), mentalization-based therapy (MBT), and transference-focused psychotherapy—are central to BPD management and have demonstrated benefit for self-harm, emotion regulation, and broader functioning [6,17]. Pharmacological approaches may still be used in BPD, but typically as adjunctive, symptom-targeted interventions rather than disorder-specific treatment [18]. In routine care, clinicians may prescribe mood stabilizers or atypical antipsychotics to target agitation, impulsiveness, aggression, insomnia, or affective lability, particularly in clinically complex presentations.

At the same time, antipsychotics and mood stabilizers are also used as augmentation strategies for depression when the response to antidepressants is limited. This creates an interpretive challenge: polypharmacy may reflect rational management of complex depression, but it may also represent attempts to manage unrecognized BPD-related dysregulation within a depression-centered formulation [18]. In such cases, labeling patients as “difficult-to-treat” may risk reinforcing a medication-centered approach, despite the possibility that prominent comorbidity (including BPD) is driving ongoing impairment and may be better addressed through revised formulation and psychotherapy-focused pathways [6].

Importantly, most evidence on BPD comorbidity relies on structured interviews or research assessments, whereas routine outpatient psychiatry units must often make decisions using time-limited documentation and prescribing histories. As a result, there is a practical need to quantify (i) how often BPD is documented and (ii) how often routine electronic medical records (EMRs) suggest possible BPD features in depressive-disorder clinics, particularly in settings where systematic PD screening is not standard practice.

Given the potential for under-recognized BPD features in Thai depression care, pragmatic strategies to support case-finding in busy outpatient services are needed. Because structured personality assessment is not routinely implemented, under-recognized BPD may nonetheless leave observable traces in routine records. In this study, we operationalized study-defined BPD indicators using information available in routine outpatient records: (1) a symptom-based indicator based on documentation of DSM-5 BPD criteria [1], and (2) a prescribing-based indicator, defined as antidepressant treatment with concurrent use of antipsychotics and/or mood stabilizers. The previous literature suggests that pharmacological management in BPD is often symptom-targeted rather than disorder-specific. Antipsychotics and mood stabilizers may be prescribed to address affective instability, impulsivity, aggression, emotional dysregulation, and associated clinical complexity commonly observed among patients with BPD. However, these medications are not specific to BPD and may also reflect augmentation strategies for difficult-to-treat depression or other psychiatric comorbidities. Therefore, in the present study, concurrent antidepressant treatment with antipsychotics and/or mood stabilizers was conceptualized as a pragmatic signal for potentially under-recognized BPD features rather than a diagnostic marker. These indicators are not substitutes for standardized diagnostic interviews and should be interpreted as prompts for further assessment rather than diagnostic confirmation. By combining symptom documentation and prescribing patterns, this approach provides a feasible, service-oriented framework for targeted case-finding using routinely collected clinical data.

Therefore, the objectives of this study were to examine the prevalence of documented BPD diagnosis recorded in the EMR and study-defined BPD indicators among adult outpatients with depressive disorders receiving pharmacological treatment at Maharaj Nakorn Chiang Mai Hospital, to quantify a subgroup who were indicator-positive in the absence of a documented diagnosis, and to describe overlap patterns between documented diagnosis, symptom-based indicators, and prescribing-based indicators to inform strategies for structured assessment, improved documentation, and access to evidence-based psychotherapy within routine depressive-disorder services.

## 2. Materials and Methods

### 2.1. Study Design

A retrospective cross-sectional study was conducted using pre-existing electronic medical records (EMRs) from Maharaj Nakorn Chiang Mai Hospital. Patient admission records included in this study spanned from January 2008 to December 2025. Although recruitment for the study sample (*n* = 1175) was conducted over a three-month period, the EMR system contains admission records dating back to 2008, and the most recent patient records included were from December 2025.

After ethics approval was obtained on 7 August 2025, EMR data extraction and chart review were conducted between September and December 2025. Clinical information was retrospectively traced back to patients’ initial clinic admissions.

### 2.2. Participants

The required sample size was calculated to estimate the prevalence of under-detected borderline personality disorder (BPD) with a specified level of precision. Using a single-proportion formula at a 95% confidence level, assuming the most conservative expected prevalence of 0.5 [19] and an absolute precision (margin of error) of ±3%, the minimum sample size was as follows:

$$n=\frac{\;Z^{2}p(1-p)}{d^{2}}=\frac{1.96^{2}\times 0.50\times 0.50}{0.03^{2}}\approx 1068$$

To account for potential exclusions and incomplete medical records, 10% inflation was applied, yielding a target sample size of 1175 patients:

Accordingly, at least 1175 eligible adult patients with depressive disorders were planned for inclusion in the retrospective review.

Inclusion criteria:

• Age ≥ 18 years;
• Diagnosis of depressive disorder (ICD-10: F32.x, F33.x, and F34.1);
• Receipt of pharmacological treatment.

Exclusion criteria:

• Psychotic depression;
• Primary psychotic disorders;
• Incomplete medical records.

### 2.3. Instruments

Data were extracted using a standardized data collection framework. The following variables were included:

• Demographic characteristics (e.g., age and sex);
• Diagnostic history (psychiatric diagnoses recorded in the EMR, including documented BPD diagnosis);
• Medication patterns (antidepressants, antipsychotics, and mood stabilizers);
• BPD features documented in clinical records, mapped to DSM-5 BPD criteria;
• Treatment outcomes (where available).

BPD indicators (study-defined): BPD-related features were identified by reviewing routine clinical documentation for text consistent with the DSM-5 BPD criteria. For analysis, the symptom-based indicator was defined as documentation of ≥5 DSM-5 BPD criteria; a threshold of ≥5 of 9 criteria was considered highly consistent with DSM-5 BPD criteria (higher-likelihood subgroup) but was not treated as a confirmed diagnosis in the absence of a structured diagnostic interview [1].

Prescribing-based indicator (study-defined): The prescribing-based indicator was defined as antidepressant treatment with concurrent use of antipsychotics and/or mood stabilizers during the study period (i.e., antidepressant plus antipsychotic and/or mood stabilizer).

### 2.4. Procedure

Data were extracted from the electronic medical records (EMRs) of the psychiatric outpatient department (OPD 24) at Maharaj Nakorn Chiang Mai Hospital between September and December 2025. Patients with depressive disorders were identified using ICD-10 codes.

Participants were categorized according to pharmacological treatment during the study period: (1) an antidepressant monotherapy group (e.g., SSRIs and/or SNRIs) and (2) a polypharmacy group defined as antidepressant treatment with concurrent use of atypical antipsychotics and/or mood stabilizers.

BPD status was ascertained from the EMR as either a documented BPD diagnosis (DSM-5/ICD-10 recorded in the chart) or study-defined BPD indicators. Within the polypharmacy group, the prescribing pattern itself constituted the prescribing-based indicator. In addition, EMR notes were reviewed for documentation consistent with DSM-5 BPD criteria to derive a symptom-based indicator of BPD features in patients without a documented diagnosis.

Where available, documentation of psychosocial interventions (e.g., psychotherapy/counseling) was extracted; however, psychotherapy records were not consistently captured in the EMR. Extracted variables were used for descriptive and comparative analyses of prevalence, clinical characteristics, and treatment patterns related to documented BPD diagnosis and indicator-positive status.

#### Operational Definitions

For the purpose of this study, **‘indicator-positive’** was defined as meeting either the symptom-based indicator (S) or the prescribing-based indicator (P) in the absence of a documented BPD diagnosis in the electronic medical records (EMR).

### 2.5. Flowchart

**Figure 1:**
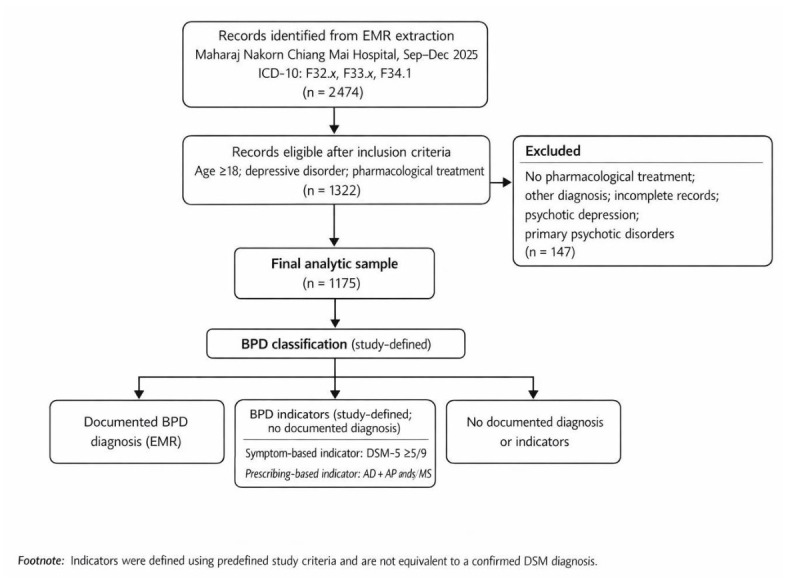
Flow diagram.

A study flowchart illustrating participant selection, grouping, and analysis procedures is provided in Figure 1.

### 2.6. Statistical Analysis

Descriptive statistics were used to summarize demographic and clinical characteristics (e.g., age, sex, and depressive-disorder subtype). Continuous variables were summarized as mean (standard deviation) or median (interquartile range), as appropriate, and categorical variables were presented as frequency (percentage). BPD status was summarized as: (1) EMR-documented BPD diagnosis and/or (2) study-defined indicators, including a symptom-based indicator (documentation of ≥5 DSM-5 BPD criteria) and a prescribing-based indicator (antidepressant treatment with concurrent antipsychotic and/or mood stabilizer use). Prevalence estimates and overlapping patterns across diagnosis and indicators were presented using a Venn distribution. All analyses were performed using SPSS version 27.

## 3. Results

The total sample consisted of 1175 patients, of whom 872 were female (74.2%) and 300 were male (25.5%). The mean age was 35.7 years (SD = 16.7), ranging from 18 to 93 years, indicating a predominantly young-to-middle-aged and female outpatient population. Regarding depressive disorder diagnoses, 749 (63.7%) had major depressive disorder (MDD), 274 (23.3%) had persistent depressive disorder (PDD), and 154 (13.0%) had other depressive disorders. A documented comorbid diagnosis of borderline personality disorder was present in 63 patients (5.4%), while 1111 (94.6%) had no recorded BPD diagnosis. All patients received an antidepressant (1175; 100%). Polypharmacy—defined as antidepressant treatment with concurrent use of antipsychotics with or without mood stabilizers—was observed in 324 patients (27.6%) (Table 1).

The distribution of BPD symptoms is presented in Table 2. Overall, 84 patients (7.1%) had documentation of ≥5 DSM-5 BPD criteria, a level highly consistent with the DSM-5 diagnostic threshold. In addition, 114 patients (9.7%) had two criteria, and 57 (4.9%), 24 (2.0%), and 32 (2.7%) patients had three, four, and five criteria, respectively, indicating a larger subgroup with subthreshold but clinically relevant BPD features based on routine clinical documentation.

**Table 2:** The distribution of symptoms for borderline personality disorder.

Number of Symptoms	*n*	%	Cumulative %
0	715	60.9	60.9
1	181	15.4	76.3
2	114	9.7	86.0
3	57	4.9	90.8
4	24	2.0	92.9
5	32	2.7	95.6
6	22	1.9	97.4
7	20	1.7	99.1
8	9	0.8	99.9
9	1	0.1	100.0

Across 1175 depressive-disorder outpatients, documentation of individual DSM-5 BPD criteria in routine clinical records ranged from 5.6% to 23.4%. The most frequently documented criterion was stress-related paranoid thoughts or severe anxiety (23.4%; 275/1175). Other commonly recorded criteria were suicidal or self-injurious behavior (13.6%; 160/1175) and intense emotional instability (13.4%; 158/1175), followed by feelings of emptiness (12.5%; 147/1175). Less frequently documented criteria included impulsive behaviors (9.4%; 111/1175), unstable interpersonal relationships (8.9%; 105/1175), inappropriate/difficult-to-control anger (7.9%; 93/1175), identity disturbance (6.3%; 74/1175), and attempts to avoid abandonment (5.6%; 66/1175) (Table 3).

**Table 3:** Prevalence of documented DSM-5 BPD criteria in routine clinical records.

BPD Symptoms	Yes	No
	*n* (%)	*n* (%)
Attempt to avoid abandonment	66 (5.6)	1109 (94.4)
Unstable interpersonal relationships	105 (8.9)	1070 (91.1)
Distortion of identity or sense of self	74 (6.3)	1101 (93.7)
Impulsive behaviors	111 (9.4)	1064 (90.6)
Suicidal or self-injurious behavior	160 (13.6)	1015 (86.4)
Intense emotional instability	158 (13.4)	1017 (86.6)
Feelings of emptiness	147 (12.5)	1028 (87.5)
Inappropriate or difficult-to-control anger	93 (7.9)	1082 (92.1)
Stress-related paranoid thoughts or severe anxiety	275 (23.4)	900 (76.6)

Most patients had no documented BPD symptoms, and documentation dropped off steeply as the number of symptoms increased. About three-quarters had 0–1 symptoms, and nearly nine in ten had 0–2 symptoms. Only a small minority had ≥5 symptoms (i.e., met the symptom-based indicator threshold), and very few had extremely high symptom counts (8–9 symptoms).

Among 1175 patients, 63 (5.4%) had a documented BPD diagnosis. Using EMR indicators, 84 patients (7.1%) met the symptom-based indicator and 325 (27.7%) met the prescribing-based indicator. In total, 374 patients (31.8%) had either a documented BPD diagnosis or at least one indicator, while 801 (68.2%) had neither. Overall, 370 (31.5%) met at least one indicator (symptom-based and/or prescribing-based). Among indicator-positive patients, 311 (84.1%) had no documented BPD diagnosis, representing 26.5% (311/1175) of the total cohort (Table 4).

**Figure 2:**
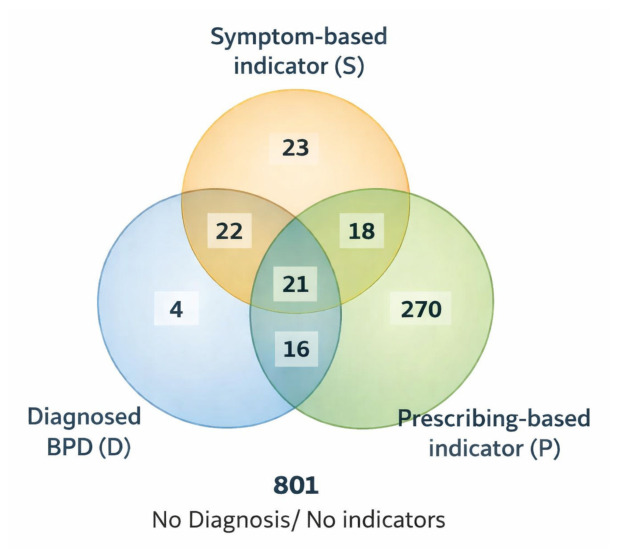
Venn distribution of documented BPD diagnosis and study-defined EMR indicators (symptom-based: ≥5 DSM-5 BPD criteria; prescribing-based: antidepressant treatment with concurrent antipsychotic and/or mood stabilizer use) among adults with depressive disorders in outpatient psychiatric care (*n* = 1175).

The Venn distribution showed 4 patients (0.3%) with a documented BPD diagnosis only, 23 (2.0%) with a symptom-based indicator only, and 270 (23.0%) with a prescribing-based indicator only. Overlap patterns included 22 (1.9%) with a documented diagnosis plus the symptom-based indicator only, 16 (1.4%) with a documented diagnosis plus the prescribing-based indicator only, 18 (1.5%) with both indicators in the absence of a documented diagnosis, and 21 (1.8%) meeting all three criteria. Patients meeting one or more indicators without a documented BPD diagnosis—considered potentially under-recognized BPD—comprised 311 of 1175 (26.5%). Among those meeting any indicator (symptom-based and/or prescribing-based; *n* = 370), 311 (84.1%) lacked a documented BPD diagnosis (Figure 2).

## 4. Discussion

This retrospective analysis highlights a substantial subgroup of patients with depressive disorders who met study-defined BPD indicators but lacked a documented BPD diagnosis. Out of 1175 individuals, 63 (5.4%) had a recorded BPD diagnosis. Overall, 363 (30.9%) met at least one study-defined indicator (symptom-based and/or prescribing-based), and 311 (26.5%) were indicator-positive without a documented diagnosis. Although these indicators do not confirm BPD in the absence of a structured diagnostic assessment, the discrepancy suggests potential under-recognition or under-documentation of BPD features in routine outpatient care. Importantly, the high proportion of patients meeting the prescribing-based indicator (antidepressant treatment with concurrent antipsychotics and/or mood stabilizers) suggests that what is treated as antidepressant nonresponse or clinical complexity in routine care may, in some cases, reflect prominent but under-recognized BPD features. In such cases, escalation or repetition of pharmacological strategies may occur in place of structured assessment and referral to BPD-informed psychotherapy.

A key clinical consideration, consistent with the concept of ‘hidden’ BPD presentations, is that BPD may be overlooked when clinicians primarily associate the disorder with highly salient crisis features such as recurrent suicidal attempts or nonsuicidal self-injury [15,20]. Zimmerman and Becker reported that a substantial proportion of outpatients meeting BPD criteria did not report recurrent self-harm yet showed functional impairment comparable to those with self-injury. This supports the view that using crisis-driven or stereotypical presentations as the primary trigger for BPD assessment may miss clinically significant cases presenting with depression, anxiety, sleep disturbance, irritability, or somatic complaints. In our sample, the large subgroup who were indicator-positive but lacked an EMR-documented BPD diagnosis may reflect a similar pathway of under-recognition, in which BPD-relevant symptoms are documented but not integrated into a recorded diagnostic formulation. In routine practice, such patients may be described as ‘difficult-to-treat’ or ‘difficult,’ which may reflect clinical complexity and comorbidity and may coexist rather than be fully explained by a medication-centered label of treatment-resistant depression.

Our findings provide a clinic-level pattern consistent with prior Thai observations that personality pathology in depressed outpatients may be under-recorded in routine services relative to rates identified using structured clinical interviews [10,11]. The large indicator-positive but undiagnosed subgroup in our sample may represent one manifestation of this documentation gap. This contrast highlights the difference between system-level diagnostic recording in routine care and structured assessment in research settings. Considered together, the large subgroup in our sample who were indicator-positive but lacked an EMR-documented BPD diagnosis provides a clinic-level signal that parallels this national-to-interview discrepancy, suggesting that clinically relevant PD/BPD features may be present but not consistently translated into routine diagnostic documentation in outpatient practice.

The distribution of overlap across indicators further informs interpretation. The largest component of the potentially under-recognized subgroup was identified by the prescribing-based indicator alone, with smaller proportions meeting the symptom-based indicator alone or meeting both indicators in the absence of an EMR-documented BPD diagnosis. This pattern suggests that clinicians may be managing distress, affective instability, impulsivity, agitation, or related pharmacological symptoms even when BPD is not formally recorded. Pascual et al. (2023) note that pharmacotherapy in BPD is typically symptom-targeted and directed toward comorbidities rather than the core disorder, and that medication may reflect overall clinical complexity [18,21]. Accordingly, prescribing patterns—particularly antipsychotic or mood stabilizer use alongside antidepressants in depressive-disorder clinics—may function as a pragmatic signal to prompt reassessment of the working formulation and evaluation for comorbid conditions, including prominent BPD features.

At the same time, medication-based indicators are inherently nonspecific, as antipsychotics may be prescribed for a wide range of symptoms and diagnoses. The previous literature suggests that antipsychotics and mood stabilizers are often prescribed to patients with BPD to target specific symptoms rather than the core disorder itself [18]. The prescribing-based indicator should therefore be interpreted as a trigger for further clinical evaluation rather than a surrogate marker of BPD. While antipsychotics and mood stabilizers can also represent evidence-based augmentation strategies for depression [22], they may additionally reflect attempts to manage complex symptom presentations that overlap with clinically significant BPD features. An exclusive focus on ‘treatment resistance’ may reinforce a medication-centered response and delay recognition of clinically significant BPD features that would warrant a different formulation and psychotherapy-oriented care pathway.

Clinically, the high proportion of patients who were indicator-positive but lacked documented BPD raises the possibility that some individuals may not be receiving BPD-informed care pathways (e.g., structured diagnostic evaluation, psychoeducation, and referral to evidence-based psychotherapy where available) [23]. More systematic screening, particularly for patients presenting with chronic affective instability, interpersonal difficulties, and recurrent functional impairment even in the absence of self-harm, may improve recognition and support more targeted, multidisciplinary management. Where available, referral to evidence-based psychotherapies may help align care with the patient’s underlying clinical needs. Future research should validate the symptom- and prescribing-based indicators against standardized diagnostic assessments to quantify their predictive performance and to clarify which patient subgroups are most likely to represent truly under-recognized BPD.

**Clinical implication**

These findings have several implications for the routine outpatient care of patients with depressive disorders. Because the indicators in this study were derived from routinely available EMR symptom documentation and prescribing data, the approach is feasible for high-throughput clinics where universal structured personality interviews are not practical. By quantifying the gap between indicator-positive status and EMR-documented diagnosis, the findings also provide a service-level baseline that can be monitored over time.

First, the large proportion of patients identified with study-defined BPD indicators but no documented diagnosis supports incorporating systematic case-finding into depressive-disorder services. A practical approach is a two-step pathway: (1) brief screening for BPD features in patients with persistent affective instability, interpersonal dysfunction, impulsivity, repeated crisis presentations, or complex medication regimens, followed by (2) structured diagnostic evaluation when screening is positive. This may reduce reliance on stereotypical presentations (e.g., self-harm) as the main trigger for considering BPD and improving diagnostic consistency.

Second, the predominance of the prescribing-based indicator subgroup suggests that medication patterns may serve as a clinical “red flag”, prompting reassessment of formulations. When antipsychotics are used in patients with depressive disorder, particularly as augmentation or for behavioral/emotional dysregulation, clinicians should consider whether the symptom profile reflects comorbidity, trauma-related presentations, bipolar spectrum illness, or prominent BPD features, and document the working diagnosis and treatment rationally and explicitly. This can help avoid prolonged, nonspecific pharmacotherapy and encourage timely referral to evidence-based psychosocial interventions. Furthermore, it should be noted that polypharmacy, especially involving antipsychotics or mood stabilizers, often unnecessarily increases the risk of side effects.

Third, improved recognition and documentation may enable more appropriate care planning, including psychoeducation, collaborative crisis planning, and referral to structured psychotherapies (where available), which are central to effective BPD management. Even when a formal diagnosis is uncertain, identifying prominent BPD features can support more tailored communication strategies, reduce iatrogenic conflict, and align treatment goals around functional recovery and safety.

Finally, from a quality-improvement perspective, the results suggest that services could monitor the gap between indicator-positive status and documented diagnosis over time as a metric of diagnostic recognition, alongside auditing prescribing practices to ensure that pharmacological treatment is symptom-targeted, time-limited where appropriate, and integrated with psychosocial care.

**Limitations**

First, using retrospective EMR data, we could not verify the duration or stability of documented BPD features; results may be influenced by diagnostic coding and documentation practices. Second, BPD status was determined using a documented EMR diagnosis and study-defined symptom- and prescribing-based indicators rather than standardized diagnostic interviews. As a result, misclassification is possible in both directions: some indicator-positive patients may not meet full BPD criteria, while some patients with BPD features may have been missed due to incomplete or inconsistent documentation.

Third, the prescribing-based indicator is inherently nonspecific. Antipsychotics and mood stabilizers may be used for a range of conditions (e.g., augmentation for treatment-resistant depression, anxiety, insomnia, irritability, or bipolar-spectrum presentations), so medication patterns should be interpreted as a prompt for further evaluation rather than evidence of BPD. Fourth, the symptom-based indicator depends on clinicians recording sufficient detail to map to DSM-5 criteria; variation in note quality, clinician focus, and structured fields could bias prevalence estimates.

Fifth, psychotherapy exposure, particularly BPD-specific interventions—such as dialectical behavior therapy (DBT), transference-focused psychotherapy, and mentalization-based therapy—was not reliably documented in the EMR dataset used for this study. Some patients may have received these interventions (within or outside the hospital) despite lacking a documented BPD diagnosis. The absence of structured psychotherapy documentation may therefore have limited our ability to identify clinically recognized BPD cases that were managed with BPD-informed psychotherapy but not coded as BPD in the diagnosis field. Incorporating a standardized psychotherapy documentation section (including therapy type and referral/attendance status) within the EMR could improve case ascertainment, clarify care pathways, and support quality improvement.

Finally, the sample was drawn from a single tertiary-care psychiatric outpatient setting over a limited time window (September–December 2025), which may limit generalizability. The study also did not validate the indicators against a gold-standard diagnostic assessment or examine longitudinal outcomes, which should be addressed in future validation and implementation studies.

## 5. Conclusions

In this psychiatric outpatient cohort of adults with depressive disorders receiving pharmacological treatment, a small proportion had an EMR-documented BPD diagnosis, whereas a substantially larger proportion were indicator-positive based on study-defined symptoms and/or prescribing indicators. This indicator-positive but undiagnosed subgroup may reflect under-recognition or under-documentation of clinically relevant BPD features in routine care and highlights an opportunity to strengthen diagnostic formulation and targeted assessment within depressive-disorder services. Interpretation is limited because EMR-based indicators are not equivalent to structured diagnostic assessment, medication-based indicators are nonspecific, and psychotherapy, particularly BPD-specific psychotherapies, was not reliably documented. Implementing systematic screening followed by structured clinical evaluation, and adding standardized EMR fields for psychotherapy referral and type, may facilitate more consistent recognition and access to BPD-informed care.

## Figures and Tables

**Table 1:** Clinical characteristics and pharmacotherapy patterns of the study cohort.

Variables	*n* (%)
Type of depressive disorder	
MDD	749 (63.70)
PDD	274 (23.30)
Other DD	154 (13.0)
Documented comorbid BPD diagnosis	
Yes	63 (5.40)
No	1111 (94.60)
Psychotropic medication	
Antidepressant	1175 (100)
Antidepressant + antipsychotic ± mood stabilizer	324 (27.60)

**Note:** Gender counts sum to 1172 (872 + 300), which is 3 fewer than the total sample (1175). This discrepancy reflects cases with missing or unknown sex information.

**Table 4:** Prevalence of documented BPD diagnosis and study-defined BPD indicators (S ≥ 5 criteria).

Category	*n* (%)
Documented BPD diagnosis (D)	63 (5.4)
Symptom-based indicator (S ≥ 5 criteria), regardless of diagnosis	84 (7.1)
Prescribing-based indicator (P), regardless of diagnosis	325 (27.7)
Any indicator (S and/or P), regardless of diagnosis	370 (31.5)
Indicator-positive without documented BPD diagnosis (I+; no D)	311 (26.5)
Prescribing-based indicator only (P only; no D)	270 (23.0)
Symptom-based indicator only (S ≥ 5 criteria; no D)	23 (2.0)
Both indicators (S + P; no D)	18 (1.5)
Indicator-positive with documented BPD diagnosis (I+ with D)	59 (5.0)
Any D/S/P (union)	374 (31.8)
No diagnosis/no indicators (None)	801 (68.2)

**Definitions.** “Symptom-based indicator (S)” was defined as documentation of ≥5 DSM-5 BPD criteria in routine clinical records. “Prescribing-based indicator (P)” was defined as antidepressant treatment with concurrent use of antipsychotics and/or mood stabilizers during the study period. “Indicator-positive” refers to meeting S and/or P in the absence of a documented BPD diagnosis.

## Data Availability

The data that support the findings of this study are not publicly available due to institutional and ethical restrictions but are available from the corresponding author upon reasonable request.

## Abbreviations

The following abbreviations are used in this manuscript:

BPD	Borderline Personality Disorder
CBT	Cognitive Behavioral Therapy
DSM-5	Diagnostic and Statistical Manual of Mental Disorders, Fifth Edition
DBT	Dialectical Behavior Therapy
EMR	Electronic Medical Record
ICD-10	International Classification of Diseases, 10th Revision
MBT	Mentalization-Based Therapy
MDD	Major Depressive Disorder
OPD	Outpatient Department
PDD	Persistent Depressive Disorder
SSRI	Selective Serotonin Reuptake Inhibitor
SNRI	Serotonin–Norepinephrine Reuptake Inhibitor

## References

[1] APA (2013). Diagnostic and Statistical Manual of Mental Disorder (DSM-5).

[2] Greenberg P., Chitnis A., Louie D., Suthoff E., Chen S.Y., Maitland J., Gagnon-Sanschagrin P., Fournier A.-A., Kessler R.C. (2023). The Economic Burden of Adults with Major Depressive Disorder in the United States (2019). Adv. Ther..

[3] Cui L., Li S., Wang S., Wu X., Liu Y., Yu W., Wang Y., Tang Y., Xia M., Li B. (2024). Major depressive disorder: Hypothesis, mechanism, prevention and treatment. Signal Transduct. Target. Ther..

[4] Chamberlain S.R., Redden S.A., Grant J.E. (2017). Associations between self-harm and distinct types of impulsivity. Psychiatry Res..

[5] Kulacaoglu F., Kose S. (2018). Borderline Personality Disorder (BPD): In the Midst of Vulnerability, Chaos, and Awe. Brain Sci..

[6] Leichsenring F., Heim N., Leweke F., Spitzer C., Steinert C., Kernberg O.F. (2023). Borderline Personality Disorder: A Review. JAMA.

[7] Gunderson J.G., Herpertz S.C., Skodol A.E., Torgersen S., Zanarini M.C. (2018). Borderline personality disorder. Nat. Rev. Dis. Prim..

[8] Rao S., Broadbear J. (2019). Borderline personality disorder and depressive disorder. Australas. Psychiatry.

[9] Vittengl J.R., Jha M.K., Minhajuddin A., Thase M.E., Jarrett R.B. (2021). Quality of life after response to acute-phase cognitive therapy for recurrent depression. J. Affect. Disord..

[10] Department of Mental Health, Ministry of Public Health (2010). Department of Mental Health, Ministry of Public Health Annual Report 2010.

[11] Wongpakaran N., Wongpakaran T., Boonyanaruthee V., Pinyopornpanish M., Intaprasert S. (2015). Comorbid personality disorders among patients with depression. Neuropsychiatr. Dis. Treat..

[12] Gunderson J.G., Stout R.L., Shea M.T., Grilo C.M., Markowitz J.C., Morey L.C., Sanislow C., Yen S., Zanarini M.C., Keuroghlian A.S. (2014). Interactions of borderline personality disorder and mood disorders over 10 years. J. Clin. Psychiatry.

[13] Gunderson J.G. (2009). Borderline personality disorder: Ontogeny of a diagnosis. Am. J. Psychiatry.

[14] Tong P., Bo P., Shi Y., Dong L., Sun T., Gao X., Yang Y. (2021). Clinical traits of patients with major depressive disorder with comorbid borderline personality disorder based on propensity score matching. Depress. Anxiety.

[15] Zimmerman M., Becker L. (2023). The hidden borderline patient: Patients with borderline personality disorder who do not engage in recurrent suicidal or self-injurious behavior. Psychol. Med..

[16] Mercer D., Douglass A., Links P. (2009). Meta-Analyses of Mood Stabilizers, Antidepressants and Antipsychotics in the Treatment of Borderline Personality Disorder: Effectiveness for Depression and Anger Symptoms. J. Personal. Disord..

[17] Bateman A., Fonagy P. (2019). A randomized controlled trial of a mentalization-based intervention (MBT-FACTS) for families of people with borderline personality disorder. Personal. Disord. Theory Res. Treat..

[18] Pascual J.C., Arias L., Soler J. (2023). Pharmacological Management of Borderline Personality Disorder and Common Comorbidities. CNS Drugs.

[19] Cochran W.G. (1963). Sampling Techniques.

[20] Wongpakaran N., Oon-Arom A., Karawekpanyawong N., Lohanan T., Leesawat T., Wongpakaran T. (2021). Borderline Personality Symptoms: What Not to Be Overlooked When Approaching Suicidal Ideation among University Students. Healthcare.

[21] Stoffers-Winterling J.M., Storebø O.J., Kongerslev M.T., Faltinsen E., Todorovac A., Sedoc Jørgensen M., Sales C.P., Callesen H.E., Ribeiro J.P., Völlm B.A. (2022). Psychotherapies for borderline personality disorder: A focused systematic review and meta-analysis. Br. J. Psychiatry.

[22] Lam R.W., Kennedy S.H., Adams C., Bahji A., Beaulieu S., Bhat V., Blier P., Blumberger D.M., Brietzke E., Chakrabarty T. (2024). Canadian Network for Mood and Anxiety Treatments (CANMAT) 2023 Update on Clinical Guidelines for Management of Major Depressive Disorder in Adults: Réseau canadien pour les traitements de l’humeur et de l’anxiété (CANMAT) 2023: Mise à jour des lignes directrices cliniques pour la prise en charge du trouble dépressif majeur chez les adultes. Can. J. Psychiatry.

[23] Cristea I.A., Gentili C., Cotet C.D., Palomba D., Barbui C., Cuijpers P. (2017). Efficacy of Psychotherapies for Borderline Personality Disorder: A Systematic Review and Meta-analysis. JAMA Psychiatry.

